# Monoclinic modification of aquadi-*n*-butyl­bis­(pyrazine-2-carboxyl­ato-κ^2^ *N* ^1^,*O*)tin(IV)

**DOI:** 10.1107/S1600536810032733

**Published:** 2010-08-21

**Authors:** Seik Weng Ng

**Affiliations:** a Department of Chemistry, University of Malaya, 50603 Kuala Lumpur, Malaysia

## Abstract

The asymmetric unit of the title organotin(IV) compound, [Sn(C_4_H_9_)_2_(C_5_H_3_N_2_O_2_)_2_(H_2_O)], contains one-and-a-half mol­ecules. The half-mol­ecule is completed by crystallographic twofold symmetry, with its Sn and water O atoms lying on the rotation axis. Both mol­ecules feature seven-coordinate Sn atoms in *trans*-C_2_SnN_2_O_3_ penta­gonal-bipyramidal coordination environments. The carboxyl­ate anions *N*,*O*-chelate to the Sn atom. In the crystal, the carboxyl­ate O atoms not involved in coordination serve as acceptors for O—H⋯O hydrogen bonds from adjacent water mol­ecules, generating a three-dimensional network.

## Related literature

For the rhombohedral modification, see: Ma *et al.* (2004 ▶).
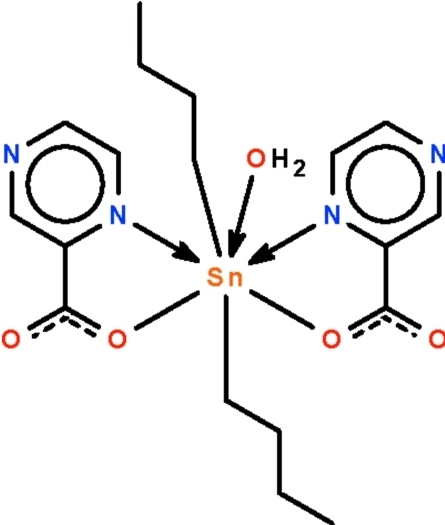


## Experimental

### 

#### Crystal data

[Sn(C_4_H_9_)_2_(C_5_H_3_N_2_O_2_)_2_(H_2_O)]
*M* *_r_* = 497.12
Monoclinic, 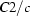

*a* = 18.8872 (9) Å
*b* = 24.4940 (11) Å
*c* = 15.4417 (7) Å
β = 119.955 (1)°
*V* = 6189.4 (5) Å^3^
*Z* = 12
Mo *K*α radiation
μ = 1.28 mm^−1^
*T* = 100 K
0.30 × 0.15 × 0.10 mm

#### Data collection

Bruker SMART APEX CCD diffractometer
Absorption correction: multi-scan (*SADABS*; Sheldrick, 1996 ▶) *T*_min_ = 0.701, *T*_max_ = 0.883
29386 measured reflections
7105 independent reflections
6558 reflections with *I* > 2σ(*I*)
*R*_int_ = 0.032

#### Refinement

*R*[*F* ^2^ > 2σ(*F* ^2^)] = 0.023
*wR*(*F* ^2^) = 0.056
*S* = 1.07
7105 reflections
392 parameters
3 restraints
H atoms treated by a mixture of independent and constrained refinement
Δρ_max_ = 0.76 e Å^−3^
Δρ_min_ = −0.50 e Å^−3^

### 

Data collection: *APEX2* (Bruker, 2009 ▶); cell refinement: *SAINT* (Bruker, 2009 ▶); data reduction: *SAINT*; program(s) used to solve structure: *SHELXS97* (Sheldrick, 2008 ▶); program(s) used to refine structure: *SHELXL97* (Sheldrick, 2008 ▶); molecular graphics: *X-SEED* (Barbour, 2001 ▶); software used to prepare material for publication: *publCIF* (Westrip, 2010 ▶).

## Supplementary Material

Crystal structure: contains datablocks global, I. DOI: 10.1107/S1600536810032733/hb5604sup1.cif

Structure factors: contains datablocks I. DOI: 10.1107/S1600536810032733/hb5604Isup2.hkl

Additional supplementary materials:  crystallographic information; 3D view; checkCIF report

## Figures and Tables

**Table 1:** Selected bond lengths (Å)

Sn1—C1	2.129 (2)
Sn1—O1	2.2590 (13)
Sn1—O1*W*	2.306 (2)
Sn1—N1	2.5333 (15)
Sn2—C10	2.124 (2)
Sn2—C14	2.128 (2)
Sn2—O5	2.2598 (13)
Sn2—O3	2.2593 (13)
Sn2—O2*W*	2.3056 (13)
Sn2—N3	2.5232 (16)
Sn2—N5	2.5362 (16)

**Table 2:** Hydrogen-bond geometry (Å, °)

*D*—H⋯*A*	*D*—H	H⋯*A*	*D*⋯*A*	*D*—H⋯*A*
O1w—H1⋯O4	0.83 (2)	1.79 (2)	2.611 (2)	170 (3)
O2w—H21⋯O2^i^	0.84 (2)	1.79 (2)	2.616 (2)	168 (2)
O2w—H22⋯O6^ii^	0.84 (2)	1.79 (2)	2.615 (2)	170 (3)

Symmetry codes: (i) 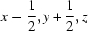; (ii) 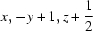.

**Fig. 1.**
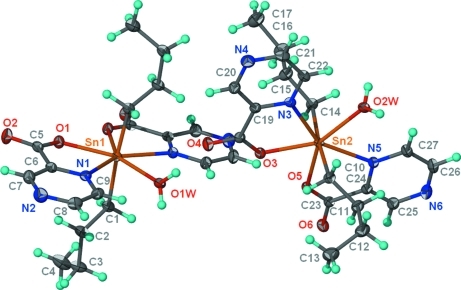
The molecular structure of (I) with displacement wllipses shown at the 70% probability level. Hydrogen atoms are drawn as spheres of arbitrary radius. One molecule lies on a twofold rotation axis; the symmetry-related atoms are not labeled.

## supplementary crystallographic information

### Comment

The title compound (I) is reported to crystallize in the rhombohedral *R*3*c* space group; the molecule lies on a twofold rotation axis that relates one alkyl group and one carboxylate anion to the other (Ma *et al.*, 2004). The present monoclinic modification features two molecules, one of which lies on a general position and the other on a twofold rotation axis. Bond dimensions between the two molecules (Fig. 1) are not significantly different, however. The molecules feature seven-coordinate tin in *trans*-C_2_SnN_2_O_3_ pentagonal bipyramidal environments. The carboxylate anions *N*,*O*-chelate to the tin atom. The carboxylate oxygen atoms not involved in coordination serve as hydrogen bond acceptor to adjacent water molecules to generate a three-dimensional hydrogen-bonded network.

### Experimental

The reaction was carried out under a nitrogen atmosphere. Pyrazine-2-carboxylic acid (0.124 g,1 mmol) and sodium ethoxide (0.068 g,1 mmol) were dissolved in to benzene (20 ml) in a Schlenk flask. Dibutyltin dichloride (0.153 g, 0.5 mmol) was added to the mixture; the mixture was stirred for 12 h at 318 K. After cooling down to the room temperature, the solution was filtered. The solvent was removed under reduced pressure to give a solid. The solid was recrystallized from ethanol to yield irregular colourless chunks of (I); yield, 72%; m.p. 517–519 K.

### Refinement

Hydrogen atoms were placed in calculated positions (C–H 0.95–0.98 Å) and included in the refinement in the riding model approximation, with *U*(H) set to 1.2–1.5*U*_eq_(C).

The water H-atoms were located in a difference Fourier map, and were refined with a distance restraint of 0.84±0.01 Å; their temperature factors were refined.

The second value in the weighting scheme is somewhat large; lowering this had only marginal impact on the refinement. The weighting scheme was the one suggested by the refinement program.

### Crystal data

**Table:**

[Sn(C_4_H_9_)_2_(C_5_H_3_N_2_O_2_)_2_(H_2_O)]	*F*(000) = 3024
*M**_r_* = 497.12	*D*_x_ = 1.600 Mg m^−^^3^
Monoclinic, *C*2/*c*	Mo *K*α radiation, λ = 0.71073 Å
Hall symbol: -C 2yc	Cell parameters from 9879 reflections
*a* = 18.8872 (9) Å	θ = 2.5–28.3°
*b* = 24.4940 (11) Å	µ = 1.28 mm^−^^1^
*c* = 15.4417 (7) Å	*T* = 100 K
β = 119.955 (1)°	Irregular, colorless
*V* = 6189.4 (5) Å^3^	0.30 × 0.15 × 0.10 mm
*Z* = 12	

### Data collection

**Table:**

Bruker SMART APEX CCD diffractometer	7105 independent reflections
Radiation source: fine-focus sealed tube	6558 reflections with *I* > 2σ(*I*)
graphite	*R*_int_ = 0.032
ω scans	θ_max_ = 27.5°, θ_min_ = 1.5°
Absorption correction: multi-scan (*SADABS*; Sheldrick, 1996)	*h* = −24→24
*T*_min_ = 0.701, *T*_max_ = 0.883	*k* = −31→28
29386 measured reflections	*l* = −20→20

### Refinement

**Table:**

Refinement on *F*^2^	Primary atom site location: structure-invariant direct methods
Least-squares matrix: full	Secondary atom site location: difference Fourier map
*R*[*F*^2^ > 2σ(*F*^2^)] = 0.023	Hydrogen site location: inferred from neighbouring sites
*wR*(*F*^2^) = 0.056	H atoms treated by a mixture of independent and constrained refinement
*S* = 1.07	*w* = 1/[σ^2^(*F*_o_^2^) + (0.021*P*)^2^ + 10.9233*P*] where *P* = (*F*_o_^2^ + 2*F*_c_^2^)/3
7105 reflections	(Δ/σ)_max_ = 0.001
392 parameters	Δρ_max_ = 0.76 e Å^−^^3^
3 restraints	Δρ_min_ = −0.50 e Å^−^^3^

### Fractional atomic coordinates and isotropic or equivalent isotropic displacement parameters (Å^2^)

**Table:**

	*x*	*y*	*z*	*U*_iso_*/*U*_eq_	
Sn1	0.5000	0.271645 (7)	0.2500	0.01226 (5)	
Sn2	0.239913 (7)	0.510838 (5)	0.272022 (9)	0.01172 (4)	
O1	0.58322 (8)	0.19810 (5)	0.29728 (10)	0.0161 (3)	
O2	0.70784 (9)	0.16107 (6)	0.38196 (11)	0.0211 (3)	
O5	0.20976 (8)	0.49615 (6)	0.11271 (10)	0.0152 (3)	
O3	0.34167 (8)	0.45819 (6)	0.28122 (10)	0.0153 (3)	
O4	0.46371 (9)	0.41771 (6)	0.37040 (10)	0.0201 (3)	
O6	0.12665 (9)	0.50371 (6)	−0.05210 (11)	0.0206 (3)	
O1W	0.5000	0.36578 (8)	0.2500	0.0174 (4)	
O2W	0.19486 (9)	0.55472 (6)	0.36767 (11)	0.0173 (3)	
N1	0.64916 (9)	0.29911 (6)	0.33560 (12)	0.0140 (3)	
N2	0.81885 (11)	0.31145 (7)	0.42537 (14)	0.0226 (4)	
N3	0.34640 (10)	0.49082 (6)	0.45089 (12)	0.0133 (3)	
N4	0.47405 (10)	0.45577 (7)	0.63621 (12)	0.0203 (4)	
N5	0.10625 (9)	0.55567 (6)	0.14732 (12)	0.0138 (3)	
N6	−0.03695 (10)	0.59800 (7)	−0.01365 (13)	0.0207 (4)	
C1	0.51096 (12)	0.27551 (8)	0.39390 (15)	0.0160 (4)	
H1A	0.5418	0.2429	0.4319	0.019*	
H1B	0.5446	0.3078	0.4288	0.019*	
C2	0.43280 (12)	0.27860 (9)	0.40007 (15)	0.0196 (4)	
H2A	0.3985	0.2463	0.3666	0.024*	
H2B	0.4016	0.3115	0.3640	0.024*	
C3	0.45043 (13)	0.28089 (9)	0.50854 (16)	0.0228 (4)	
H3A	0.4890	0.3112	0.5436	0.027*	
H3B	0.3989	0.2891	0.5081	0.027*	
C4	0.48623 (15)	0.22850 (10)	0.56649 (18)	0.0304 (5)	
H4A	0.4958	0.2327	0.6346	0.046*	
H4B	0.5381	0.2206	0.5690	0.046*	
H4C	0.4479	0.1984	0.5333	0.046*	
C5	0.66054 (11)	0.20028 (8)	0.34647 (14)	0.0149 (4)	
C6	0.69917 (11)	0.25600 (8)	0.36406 (14)	0.0145 (4)	
C7	0.78340 (12)	0.26246 (8)	0.40881 (15)	0.0187 (4)	
H7	0.8170	0.2308	0.4283	0.022*	
C8	0.76820 (12)	0.35409 (9)	0.39651 (15)	0.0197 (4)	
H8	0.7906	0.3898	0.4066	0.024*	
C9	0.68399 (12)	0.34850 (8)	0.35234 (15)	0.0172 (4)	
H9	0.6506	0.3802	0.3337	0.021*	
C10	0.30749 (11)	0.58260 (8)	0.28432 (14)	0.0155 (4)	
H10A	0.3269	0.5984	0.3515	0.019*	
H10B	0.3564	0.5717	0.2807	0.019*	
C11	0.26372 (12)	0.62761 (8)	0.20726 (16)	0.0188 (4)	
H11A	0.2157	0.6401	0.2116	0.023*	
H11B	0.2436	0.6125	0.1394	0.023*	
C12	0.31919 (13)	0.67654 (9)	0.22259 (17)	0.0233 (4)	
H12A	0.2856	0.7067	0.1785	0.028*	
H12B	0.3433	0.6893	0.2925	0.028*	
C13	0.38749 (14)	0.66419 (10)	0.20121 (19)	0.0304 (5)	
H13A	0.4204	0.6971	0.2122	0.046*	
H13B	0.3642	0.6523	0.1316	0.046*	
H13C	0.4221	0.6351	0.2459	0.046*	
C14	0.16848 (11)	0.44272 (8)	0.26816 (14)	0.0150 (4)	
H14A	0.1206	0.4567	0.2711	0.018*	
H14B	0.1476	0.4244	0.2027	0.018*	
C15	0.21022 (12)	0.39971 (8)	0.34983 (15)	0.0185 (4)	
H15A	0.2271	0.4166	0.4156	0.022*	
H15B	0.2601	0.3866	0.3505	0.022*	
C16	0.15458 (12)	0.35109 (8)	0.33474 (16)	0.0213 (4)	
H16A	0.1041	0.3642	0.3325	0.026*	
H16B	0.1388	0.3335	0.2698	0.026*	
C17	0.19607 (14)	0.30907 (9)	0.41807 (18)	0.0273 (5)	
H17A	0.1585	0.2787	0.4059	0.041*	
H17B	0.2109	0.3262	0.4823	0.041*	
H17C	0.2454	0.2954	0.4196	0.041*	
C18	0.40452 (11)	0.44366 (8)	0.36248 (14)	0.0141 (4)	
C19	0.40703 (11)	0.45937 (7)	0.45834 (14)	0.0134 (4)	
C20	0.47023 (12)	0.44212 (8)	0.55007 (14)	0.0169 (4)	
H20	0.5122	0.4199	0.5518	0.020*	
C21	0.41298 (13)	0.48679 (8)	0.62802 (15)	0.0193 (4)	
H21A	0.4130	0.4972	0.6873	0.023*	
C22	0.34938 (12)	0.50456 (8)	0.53674 (15)	0.0161 (4)	
H22A	0.3074	0.5267	0.5351	0.019*	
C23	0.14592 (11)	0.51309 (8)	0.03584 (14)	0.0144 (4)	
C24	0.08750 (11)	0.54742 (8)	0.05262 (14)	0.0136 (4)	
C25	0.01660 (12)	0.56846 (8)	−0.02682 (15)	0.0175 (4)	
H25	0.0056	0.5617	−0.0930	0.021*	
C26	−0.01696 (12)	0.60683 (8)	0.08106 (16)	0.0197 (4)	
H26	−0.0525	0.6283	0.0941	0.024*	
C27	0.05379 (12)	0.58593 (8)	0.16149 (15)	0.0175 (4)	
H27	0.0651	0.5932	0.2276	0.021*	
H1	0.4900 (17)	0.3856 (10)	0.2865 (18)	0.035 (7)*	
H21	0.1969 (15)	0.5884 (7)	0.3783 (18)	0.023 (6)*	
H22	0.1695 (16)	0.5391 (11)	0.392 (2)	0.036 (8)*	

### Atomic displacement parameters (Å^2^)

**Table:**

	*U*^11^	*U*^22^	*U*^33^	*U*^12^	*U*^13^	*U*^23^
Sn1	0.01380 (9)	0.01078 (9)	0.01431 (9)	0.000	0.00861 (7)	0.000
Sn2	0.01378 (6)	0.01148 (7)	0.01148 (7)	0.00100 (5)	0.00750 (5)	0.00075 (4)
O1	0.0156 (6)	0.0120 (6)	0.0214 (7)	−0.0005 (5)	0.0098 (6)	−0.0008 (5)
O2	0.0192 (7)	0.0133 (7)	0.0297 (8)	0.0028 (6)	0.0113 (6)	0.0005 (6)
O5	0.0153 (6)	0.0182 (7)	0.0129 (7)	0.0039 (5)	0.0077 (5)	0.0007 (5)
O3	0.0165 (6)	0.0171 (7)	0.0129 (6)	0.0032 (5)	0.0078 (5)	0.0010 (5)
O4	0.0201 (7)	0.0234 (8)	0.0163 (7)	0.0076 (6)	0.0088 (6)	0.0002 (6)
O6	0.0226 (7)	0.0264 (8)	0.0140 (7)	0.0073 (6)	0.0101 (6)	0.0009 (6)
O1W	0.0261 (10)	0.0114 (9)	0.0230 (11)	0.000	0.0185 (9)	0.000
O2W	0.0259 (7)	0.0129 (7)	0.0204 (7)	0.0005 (6)	0.0171 (6)	0.0002 (6)
N1	0.0156 (7)	0.0131 (8)	0.0142 (8)	−0.0011 (6)	0.0081 (6)	0.0002 (6)
N2	0.0188 (8)	0.0228 (9)	0.0262 (10)	−0.0029 (7)	0.0112 (8)	−0.0019 (7)
N3	0.0150 (7)	0.0137 (8)	0.0121 (8)	−0.0015 (6)	0.0076 (6)	−0.0005 (6)
N4	0.0207 (8)	0.0243 (9)	0.0141 (8)	0.0004 (7)	0.0073 (7)	0.0008 (7)
N5	0.0139 (7)	0.0132 (8)	0.0150 (8)	0.0003 (6)	0.0079 (6)	−0.0002 (6)
N6	0.0176 (8)	0.0201 (9)	0.0221 (9)	0.0046 (7)	0.0082 (7)	0.0015 (7)
C1	0.0172 (9)	0.0173 (10)	0.0150 (9)	0.0005 (7)	0.0092 (8)	0.0008 (7)
C2	0.0185 (9)	0.0259 (11)	0.0176 (10)	0.0037 (8)	0.0115 (8)	0.0034 (8)
C3	0.0261 (11)	0.0272 (11)	0.0207 (10)	0.0049 (9)	0.0160 (9)	0.0015 (9)
C4	0.0325 (12)	0.0380 (14)	0.0298 (12)	0.0097 (10)	0.0225 (11)	0.0102 (10)
C5	0.0170 (9)	0.0151 (9)	0.0159 (9)	−0.0003 (7)	0.0106 (8)	−0.0016 (7)
C6	0.0164 (9)	0.0156 (9)	0.0138 (9)	−0.0005 (7)	0.0092 (7)	−0.0010 (7)
C7	0.0161 (9)	0.0176 (10)	0.0222 (10)	0.0001 (7)	0.0095 (8)	−0.0006 (8)
C8	0.0200 (10)	0.0178 (10)	0.0210 (10)	−0.0056 (8)	0.0099 (8)	−0.0010 (8)
C9	0.0202 (9)	0.0139 (9)	0.0177 (10)	−0.0021 (7)	0.0096 (8)	−0.0001 (7)
C10	0.0156 (9)	0.0152 (9)	0.0153 (9)	−0.0003 (7)	0.0074 (7)	0.0012 (7)
C11	0.0172 (9)	0.0173 (10)	0.0221 (10)	0.0006 (7)	0.0099 (8)	0.0047 (8)
C12	0.0236 (10)	0.0176 (10)	0.0294 (12)	−0.0025 (8)	0.0139 (9)	0.0052 (9)
C13	0.0238 (11)	0.0314 (13)	0.0398 (14)	−0.0001 (9)	0.0188 (10)	0.0135 (11)
C14	0.0138 (8)	0.0148 (9)	0.0154 (9)	−0.0013 (7)	0.0065 (7)	−0.0002 (7)
C15	0.0185 (9)	0.0152 (9)	0.0181 (10)	−0.0031 (7)	0.0065 (8)	0.0010 (8)
C16	0.0192 (9)	0.0175 (10)	0.0235 (11)	−0.0048 (8)	0.0078 (8)	0.0014 (8)
C17	0.0302 (12)	0.0187 (11)	0.0303 (12)	−0.0039 (9)	0.0130 (10)	0.0035 (9)
C18	0.0169 (9)	0.0116 (9)	0.0147 (9)	−0.0007 (7)	0.0086 (8)	−0.0008 (7)
C19	0.0146 (8)	0.0115 (9)	0.0149 (9)	−0.0023 (7)	0.0081 (7)	−0.0004 (7)
C20	0.0170 (9)	0.0171 (10)	0.0152 (9)	0.0016 (7)	0.0071 (8)	0.0006 (7)
C21	0.0225 (10)	0.0228 (10)	0.0135 (9)	−0.0019 (8)	0.0097 (8)	−0.0014 (8)
C22	0.0169 (9)	0.0181 (9)	0.0157 (9)	−0.0017 (7)	0.0099 (8)	−0.0025 (7)
C23	0.0159 (9)	0.0134 (9)	0.0158 (9)	0.0010 (7)	0.0092 (8)	0.0010 (7)
C24	0.0146 (8)	0.0114 (9)	0.0157 (9)	−0.0017 (7)	0.0083 (7)	0.0000 (7)
C25	0.0173 (9)	0.0184 (10)	0.0163 (9)	0.0013 (7)	0.0079 (8)	0.0007 (8)
C26	0.0176 (9)	0.0184 (10)	0.0249 (11)	0.0024 (8)	0.0121 (8)	−0.0017 (8)
C27	0.0179 (9)	0.0178 (10)	0.0193 (10)	0.0010 (8)	0.0112 (8)	−0.0018 (8)

### Geometric parameters (Å, °)

**Table:**

Sn1—C1	2.129 (2)	C4—H4B	0.9800
Sn1—C1^i^	2.129 (2)	C4—H4C	0.9800
Sn1—O1^i^	2.2590 (13)	C5—C6	1.507 (3)
Sn1—O1	2.2590 (13)	C6—C7	1.391 (3)
Sn1—O1W	2.306 (2)	C7—H7	0.9500
Sn1—N1	2.5333 (15)	C8—C9	1.389 (3)
Sn1—N1^i^	2.5333 (15)	C8—H8	0.9500
Sn2—C10	2.124 (2)	C9—H9	0.9500
Sn2—C14	2.128 (2)	C10—C11	1.527 (3)
Sn2—O5	2.2598 (13)	C10—H10A	0.9900
Sn2—O3	2.2593 (13)	C10—H10B	0.9900
Sn2—O2W	2.3056 (13)	C11—C12	1.530 (3)
Sn2—N3	2.5232 (16)	C11—H11A	0.9900
Sn2—N5	2.5362 (16)	C11—H11B	0.9900
O1—C5	1.267 (2)	C12—C13	1.513 (3)
O2—C5	1.238 (2)	C12—H12A	0.9900
O5—C23	1.267 (2)	C12—H12B	0.9900
O3—C18	1.274 (2)	C13—H13A	0.9800
O4—C18	1.238 (2)	C13—H13B	0.9800
O6—C23	1.239 (2)	C13—H13C	0.9800
O1W—H1	0.833 (16)	C14—C15	1.526 (3)
O2W—H21	0.839 (16)	C14—H14A	0.9900
O2W—H22	0.838 (17)	C14—H14B	0.9900
N1—C6	1.336 (2)	C15—C16	1.527 (3)
N1—C9	1.339 (2)	C15—H15A	0.9900
N2—C8	1.334 (3)	C15—H15B	0.9900
N2—C7	1.335 (3)	C16—C17	1.524 (3)
N3—C19	1.337 (2)	C16—H16A	0.9900
N3—C22	1.341 (2)	C16—H16B	0.9900
N4—C21	1.334 (3)	C17—H17A	0.9800
N4—C20	1.338 (3)	C17—H17B	0.9800
N5—C24	1.337 (2)	C17—H17C	0.9800
N5—C27	1.340 (2)	C18—C19	1.507 (3)
N6—C26	1.333 (3)	C19—C20	1.386 (3)
N6—C25	1.339 (3)	C20—H20	0.9500
C1—C2	1.528 (3)	C21—C22	1.389 (3)
C1—H1A	0.9900	C21—H21A	0.9500
C1—H1B	0.9900	C22—H22A	0.9500
C2—C3	1.537 (3)	C23—C24	1.509 (3)
C2—H2A	0.9900	C24—C25	1.387 (3)
C2—H2B	0.9900	C25—H25	0.9500
C3—C4	1.517 (3)	C26—C27	1.390 (3)
C3—H3A	0.9900	C26—H26	0.9500
C3—H3B	0.9900	C27—H27	0.9500
C4—H4A	0.9800		
			
C1—Sn1—C1^i^	174.9 (1)	N1—C6—C5	117.21 (16)
C1—Sn1—O1^i^	93.56 (6)	C7—C6—C5	121.55 (17)
C1^i^—Sn1—O1^i^	90.50 (6)	N2—C7—C6	122.49 (19)
C1—Sn1—O1	90.50 (6)	N2—C7—H7	118.8
C1^i^—Sn1—O1	93.56 (6)	C6—C7—H7	118.8
O1^i^—Sn1—O1	74.22 (7)	N2—C8—C9	122.76 (19)
C1—Sn1—O1W	87.45 (5)	N2—C8—H8	118.6
C1^i^—Sn1—O1W	87.45 (5)	C9—C8—H8	118.6
O1^i^—Sn1—O1W	142.89 (3)	N1—C9—C8	121.03 (19)
O1—Sn1—O1W	142.89 (3)	N1—C9—H9	119.5
C1—Sn1—N1	86.58 (6)	C8—C9—H9	119.5
C1^i^—Sn1—N1	92.07 (6)	C11—C10—Sn2	117.40 (13)
O1^i^—Sn1—N1	142.51 (5)	C11—C10—H10A	108.0
O1—Sn1—N1	68.29 (5)	Sn2—C10—H10A	108.0
O1W—Sn1—N1	74.60 (4)	C11—C10—H10B	108.0
C1—Sn1—N1^i^	92.07 (6)	Sn2—C10—H10B	108.0
C1^i^—Sn1—N1^i^	86.58 (6)	H10A—C10—H10B	107.2
O1^i^—Sn1—N1^i^	68.29 (5)	C10—C11—C12	112.47 (17)
O1—Sn1—N1^i^	142.51 (5)	C10—C11—H11A	109.1
O1W—Sn1—N1^i^	74.60 (4)	C12—C11—H11A	109.1
N1—Sn1—N1^i^	149.20 (7)	C10—C11—H11B	109.1
C10—Sn2—C14	174.6 (1)	C12—C11—H11B	109.1
C10—Sn2—O5	92.44 (6)	H11A—C11—H11B	107.8
C14—Sn2—O5	91.90 (6)	C13—C12—C11	113.71 (19)
C10—Sn2—O3	90.71 (6)	C13—C12—H12A	108.8
C14—Sn2—O3	93.54 (6)	C11—C12—H12A	108.8
O5—Sn2—O3	73.84 (5)	C13—C12—H12B	108.8
C10—Sn2—O2W	87.39 (6)	C11—C12—H12B	108.8
C14—Sn2—O2W	87.24 (6)	H12A—C12—H12B	107.7
O5—Sn2—O2W	143.00 (5)	C12—C13—H13A	109.5
O3—Sn2—O2W	143.16 (5)	C12—C13—H13B	109.5
C10—Sn2—N3	87.01 (6)	H13A—C13—H13B	109.5
C14—Sn2—N3	91.49 (6)	C12—C13—H13C	109.5
O5—Sn2—N3	142.24 (5)	H13A—C13—H13C	109.5
O3—Sn2—N3	68.42 (5)	H13B—C13—H13C	109.5
O2W—Sn2—N3	74.74 (5)	C15—C14—Sn2	117.36 (13)
C10—Sn2—N5	92.04 (6)	C15—C14—H14A	108.0
C14—Sn2—N5	86.64 (6)	Sn2—C14—H14A	108.0
O5—Sn2—N5	68.17 (5)	C15—C14—H14B	108.0
O3—Sn2—N5	141.98 (5)	Sn2—C14—H14B	108.0
O2W—Sn2—N5	74.85 (5)	H14A—C14—H14B	107.2
N3—Sn2—N5	149.59 (5)	C14—C15—C16	112.34 (16)
C5—O1—Sn1	124.61 (12)	C14—C15—H15A	109.1
C23—O5—Sn2	124.90 (12)	C16—C15—H15A	109.1
C18—O3—Sn2	124.50 (12)	C14—C15—H15B	109.1
Sn1—O1W—H1	125.6 (19)	C16—C15—H15B	109.1
Sn2—O2W—H21	126.0 (17)	H15A—C15—H15B	107.9
Sn2—O2W—H22	124 (2)	C17—C16—C15	111.92 (17)
H21—O2W—H22	110 (3)	C17—C16—H16A	109.2
C6—N1—C9	116.87 (16)	C15—C16—H16A	109.2
C6—N1—Sn1	112.39 (12)	C17—C16—H16B	109.2
C9—N1—Sn1	130.68 (13)	C15—C16—H16B	109.2
C8—N2—C7	115.62 (17)	H16A—C16—H16B	107.9
C19—N3—C22	116.63 (16)	C16—C17—H17A	109.5
C19—N3—Sn2	112.74 (12)	C16—C17—H17B	109.5
C22—N3—Sn2	130.56 (13)	H17A—C17—H17B	109.5
C21—N4—C20	115.68 (17)	C16—C17—H17C	109.5
C24—N5—C27	116.65 (17)	H17A—C17—H17C	109.5
C24—N5—Sn2	112.63 (12)	H17B—C17—H17C	109.5
C27—N5—Sn2	130.72 (13)	O4—C18—O3	126.31 (18)
C26—N6—C25	115.64 (17)	O4—C18—C19	116.75 (17)
C2—C1—Sn1	118.29 (13)	O3—C18—C19	116.94 (16)
C2—C1—H1A	107.7	N3—C19—C20	121.82 (17)
Sn1—C1—H1A	107.7	N3—C19—C18	117.20 (16)
C2—C1—H1B	107.7	C20—C19—C18	120.98 (17)
Sn1—C1—H1B	107.7	N4—C20—C19	122.09 (18)
H1A—C1—H1B	107.1	N4—C20—H20	119.0
C1—C2—C3	112.30 (17)	C19—C20—H20	119.0
C1—C2—H2A	109.1	N4—C21—C22	122.94 (18)
C3—C2—H2A	109.1	N4—C21—H21A	118.5
C1—C2—H2B	109.1	C22—C21—H21A	118.5
C3—C2—H2B	109.1	N3—C22—C21	120.84 (18)
H2A—C2—H2B	107.9	N3—C22—H22A	119.6
C4—C3—C2	113.71 (18)	C21—C22—H22A	119.6
C4—C3—H3A	108.8	O6—C23—O5	125.95 (17)
C2—C3—H3A	108.8	O6—C23—C24	116.85 (17)
C4—C3—H3B	108.8	O5—C23—C24	117.20 (17)
C2—C3—H3B	108.8	N5—C24—C25	121.52 (17)
H3A—C3—H3B	107.7	N5—C24—C23	117.05 (16)
C3—C4—H4A	109.5	C25—C24—C23	121.41 (17)
C3—C4—H4B	109.5	N6—C25—C24	122.37 (18)
H4A—C4—H4B	109.5	N6—C25—H25	118.8
C3—C4—H4C	109.5	C24—C25—H25	118.8
H4A—C4—H4C	109.5	N6—C26—C27	122.66 (18)
H4B—C4—H4C	109.5	N6—C26—H26	118.7
O2—C5—O1	126.44 (18)	C27—C26—H26	118.7
O2—C5—C6	116.45 (17)	N5—C27—C26	121.14 (18)
O1—C5—C6	117.11 (17)	N5—C27—H27	119.4
N1—C6—C7	121.23 (18)	C26—C27—H27	119.4
			
C1—Sn1—O1—C5	−81.98 (15)	C9—N1—C6—C7	−0.1 (3)
C1^i^—Sn1—O1—C5	94.95 (15)	Sn1—N1—C6—C7	177.36 (14)
O1^i^—Sn1—O1—C5	−175.54 (17)	C9—N1—C6—C5	179.39 (16)
O1W—Sn1—O1—C5	4.46 (17)	Sn1—N1—C6—C5	−3.17 (19)
N1—Sn1—O1—C5	4.14 (14)	O2—C5—C6—N1	−173.10 (17)
N1^i^—Sn1—O1—C5	−176.03 (13)	O1—C5—C6—N1	6.7 (2)
C10—Sn2—O5—C23	−92.57 (15)	O2—C5—C6—C7	6.4 (3)
C14—Sn2—O5—C23	84.26 (15)	O1—C5—C6—C7	−173.81 (17)
O3—Sn2—O5—C23	177.40 (16)	C8—N2—C7—C6	0.2 (3)
O2W—Sn2—O5—C23	−3.67 (19)	N1—C6—C7—N2	−0.3 (3)
N3—Sn2—O5—C23	179.15 (13)	C5—C6—C7—N2	−179.77 (18)
N5—Sn2—O5—C23	−1.36 (14)	C7—N2—C8—C9	0.2 (3)
C10—Sn2—O3—C18	83.97 (15)	C6—N1—C9—C8	0.5 (3)
C14—Sn2—O3—C18	−92.74 (15)	Sn1—N1—C9—C8	−176.36 (14)
O5—Sn2—O3—C18	176.31 (15)	N2—C8—C9—N1	−0.6 (3)
O2W—Sn2—O3—C18	−2.62 (19)	O5—Sn2—C10—C11	55.92 (15)
N3—Sn2—O3—C18	−2.54 (14)	O3—Sn2—C10—C11	129.77 (14)
N5—Sn2—O3—C18	178.18 (13)	O2W—Sn2—C10—C11	−87.04 (15)
C1—Sn1—N1—C6	91.88 (13)	N3—Sn2—C10—C11	−161.89 (15)
C1^i^—Sn1—N1—C6	−93.04 (13)	N5—Sn2—C10—C11	−12.31 (15)
O1^i^—Sn1—N1—C6	0.48 (17)	Sn2—C10—C11—C12	−178.77 (14)
O1—Sn1—N1—C6	−0.03 (12)	C10—C11—C12—C13	68.0 (2)
O1W—Sn1—N1—C6	−179.83 (13)	O5—Sn2—C14—C15	125.86 (14)
N1^i^—Sn1—N1—C6	−179.83 (13)	O3—Sn2—C14—C15	51.94 (14)
C1—Sn1—N1—C9	−91.14 (17)	O2W—Sn2—C14—C15	−91.17 (14)
C1^i^—Sn1—N1—C9	83.95 (17)	N3—Sn2—C14—C15	−16.53 (15)
O1^i^—Sn1—N1—C9	177.47 (14)	N5—Sn2—C14—C15	−166.15 (15)
O1—Sn1—N1—C9	176.96 (18)	Sn2—C14—C15—C16	−175.97 (13)
O1W—Sn1—N1—C9	−2.84 (15)	C14—C15—C16—C17	−178.56 (18)
N1^i^—Sn1—N1—C9	−2.84 (15)	Sn2—O3—C18—O4	−175.31 (15)
C10—Sn2—N3—C19	−92.41 (13)	Sn2—O3—C18—C19	4.9 (2)
C14—Sn2—N3—C19	92.76 (13)	C22—N3—C19—C20	−0.5 (3)
O5—Sn2—N3—C19	−2.27 (17)	Sn2—N3—C19—C20	−177.75 (14)
O3—Sn2—N3—C19	−0.46 (12)	C22—N3—C19—C18	−179.94 (16)
O2W—Sn2—N3—C19	179.49 (13)	Sn2—N3—C19—C18	2.8 (2)
N5—Sn2—N3—C19	178.66 (11)	O4—C18—C19—N3	175.18 (17)
C10—Sn2—N3—C22	90.84 (17)	O3—C18—C19—N3	−5.0 (2)
C14—Sn2—N3—C22	−84.00 (17)	O4—C18—C19—C20	−4.3 (3)
O5—Sn2—N3—C22	−179.03 (14)	O3—C18—C19—C20	175.54 (17)
O3—Sn2—N3—C22	−177.22 (17)	C21—N4—C20—C19	0.3 (3)
O2W—Sn2—N3—C22	2.73 (16)	N3—C19—C20—N4	0.2 (3)
N5—Sn2—N3—C22	1.9 (2)	C18—C19—C20—N4	179.64 (18)
C10—Sn2—N5—C24	91.76 (13)	C20—N4—C21—C22	−0.5 (3)
C14—Sn2—N5—C24	−93.45 (13)	C19—N3—C22—C21	0.3 (3)
O5—Sn2—N5—C24	−0.05 (12)	Sn2—N3—C22—C21	176.94 (14)
O3—Sn2—N5—C24	−1.99 (17)	N4—C21—C22—N3	0.3 (3)
O2W—Sn2—N5—C24	178.51 (13)	Sn2—O5—C23—O6	−176.99 (15)
N3—Sn2—N5—C24	179.33 (11)	Sn2—O5—C23—C24	2.4 (2)
C10—Sn2—N5—C27	−87.34 (17)	C27—N5—C24—C25	−1.0 (3)
C14—Sn2—N5—C27	87.45 (17)	Sn2—N5—C24—C25	179.77 (14)
O5—Sn2—N5—C27	−179.16 (18)	C27—N5—C24—C23	−179.62 (16)
O3—Sn2—N5—C27	178.90 (14)	Sn2—N5—C24—C23	1.1 (2)
O2W—Sn2—N5—C27	−0.60 (16)	O6—C23—C24—N5	177.18 (17)
N3—Sn2—N5—C27	0.2 (2)	O5—C23—C24—N5	−2.3 (3)
O1^i^—Sn1—C1—C2	−54.71 (15)	O6—C23—C24—C25	−1.4 (3)
O1—Sn1—C1—C2	−128.93 (15)	O5—C23—C24—C25	179.07 (18)
O1W—Sn1—C1—C2	88.14 (15)	C26—N6—C25—C24	1.3 (3)
N1—Sn1—C1—C2	162.85 (15)	N5—C24—C25—N6	−0.1 (3)
N1^i^—Sn1—C1—C2	13.66 (15)	C23—C24—C25—N6	178.49 (18)
Sn1—C1—C2—C3	179.73 (14)	C25—N6—C26—C27	−1.4 (3)
C1—C2—C3—C4	−68.0 (2)	C24—N5—C27—C26	0.9 (3)
Sn1—O1—C5—O2	172.50 (15)	Sn2—N5—C27—C26	179.93 (14)
Sn1—O1—C5—C6	−7.3 (2)	N6—C26—C27—N5	0.4 (3)

Symmetry codes: (i) −*x*+1, *y*, −*z*+1/2.

### Hydrogen-bond geometry (Å, °)

**Table:**

*D*—H···*A*	*D*—H	H···*A*	*D*···*A*	*D*—H···*A*
O1w—H1···O4	0.83 (2)	1.79 (2)	2.611 (2)	170 (3)
O2w—H21···O2^ii^	0.84 (2)	1.79 (2)	2.616 (2)	168 (2)
O2w—H22···O6^iii^	0.84 (2)	1.79 (2)	2.615 (2)	170 (3)

Symmetry codes: (ii) *x*−1/2, *y*+1/2, *z*; (iii) *x*, −*y*+1, *z*+1/2.
